# A Comprehensive Review on Stability Analysis of Hybrid Energy System

**DOI:** 10.3390/s25102974

**Published:** 2025-05-08

**Authors:** Namita Kumari, Binh Tran, Ankush Sharma, Damminda Alahakoon

**Affiliations:** 1 Department of Electrical Engineering, Indian Institute of Technology, Kanpur 208016, India; nkumari21@iitk.ac.in (N.K.); ansharma@iitk.ac.in (A.S.); 2 Centre for Data Analytics and Cognition, La Trobe University, Melbourne, VIC 3083, Australia; b.tran@latrobe.edu.au

**Keywords:** hybrid energy system, renewable energy, stability analysis

## Abstract

Hybrid Energy Systems (HES) are pivotal in modern power systems. They incorporate conventional and renewable energy sources, energy storage, and main grids to deliver reliable and sustainable power. To ensure the smooth functioning of such systems, stability analysis is essential, particularly in dynamic and unpredictable situations. Despite tremendous progress, the stability analysis of HES is still complex due to challenges such as nonlinearity, system complexity, and uncertainty in renewable energy generation. A thorough understanding of stability analysis for HES is crucial to ensure the reliable and efficient design of these complex power systems. Particularly in the current data-intensive era, vast volumes of data are being collected through advanced sensors and communication technologies. However, no thorough and organised discussion of every facet of HES stability analysis is available in the literature. This paper aims to review various types and techniques for analysing frequency, transient, small-signal, and converter-driven stability, and to assess the importance and challenges of such analyses for HES. By emphasising the need for innovative approaches for stability enhancement, the paper also discusses the importance of continued research in optimising the operation and reliability of hybrid energy systems.

## 1. Introduction

The growing concerns regarding energy sustainability and reliability have intensified due to the depletion of traditional fossil fuels and the intermittent and non-dispatchable nature of renewable energy resources. In addition, as populations and industrialization expand and drive global energy consumption, traditional energy sources face problems such as increased costs, supply chain weaknesses, and environmental concerns such as climate change. However, renewable energy sources such as solar, wind, hydro and biomass provide an abundant, sustainable, and clean energy alternative, minimizing reliance on traditional sources of energy while maintaining supply–demand balance. However, managing the interactions between many energy sources has resulted in the creation of Hybrid Energy Systems (HESs), which seek to deliver sustainable, reliable, and cost-effective energy solutions.

In general terms, the HES is characterized as a power generation system that consists of at least two distinct energy mechanisms that operate on different energy resources to enhance the reliability of the power supply. This energy system can occasionally consist of three or four distinct energy sources powered by various technologies, including diesel generators, geothermal, modest hydropower, solar photovoltaic systems, and wind energy conversion systems. HES was created to benefit from both conventional and unconventional energy sources [1], while addressing the limitations of each other. This combination addresses the intermittent nature of renewable energy sources, minimizes greenhouse gas emissions, and ensures a reliable power supply [2]. Decentralized energy generation through HESs also improves resilience, reduces dependence on centralized grids, and benefits remote locations where grid extension is not practical. HESs are usually preferred in isolated and off-grid locations with little to no connection to central utility systems. The use of HESs has been further expanded when combining different energy sources with energy storage. They provide a steady power supply by fusing energy storage with renewable [3,4] and conventional energy sources.

HESs help industries meet their high energy needs while reducing their carbon footprints [5]. By incorporating renewable energy sources, HESs increase energy efficiency and guarantee energy supply during periods of high demand. HESs have shown their importance in meeting energy demands in various fields [6]. Applications for HESs are varied and span multiple sectors, especially in areas with unreliable grid access. Solar–diesel and wind–diesel combinations are very efficient for irrigation and water pumping in agriculture. Additionally, HESs are also used for commercial and institutional buildings and purposes, including offices, hospitals, transportation and schools, guaranteeing a continuous power supply while lowering reliance on traditional energy sources [7].

Control techniques [8] are necessary to manage and maintain the power system to operate efficiently alongside both conventional and renewable energy resources. They need complex control strategies [9], energy management systems (EMSs) [10], and a strong communication [11] network to coordinate different power sources, including conventional and renewable energy. The complicated nature of HESs makes control, stability, and reliability management difficult. Conventional methods of mathematical control techniques are often laborious and time-consuming. However, because of technological improvements and the availability of data-gathering technologies like sensors, smart meters, and Phasor Measurement Units (PMUs) [12], it has become much simpler and faster to develop robust control techniques and other assessments.

Traditional mathematical control techniques are very rigorous and time-consuming. However, with advancement in technologies and data-gathering tools, such as sensors, smart meters and Phasor Measurement Units (PMUs) [12], it is easier to carry out thorough and fast analyses on these important topics. These technologies facilitate improved management and optimization of HESs by enabling real-time monitoring, accurate measurements, and a deeper understanding of system dynamics. HESs can work independently as well as in the network; these control systems are required to regulate fluctuations in the power supply.

The intermittent nature of renewable energy makes HES more prone to instability. Dynamic interactions between components, arising from the integration of various energy sources and energy storage systems [6], add complexity to HES stability. Potential issues, including load variations, power supply fluctuations, and the effects of intermittent renewable energy sources, can be identified and mitigated with stability analysis [13]. The performance and safety of the system may be jeopardized in the absence of stability due to operational problems such as voltage instability, frequency variations, or inefficient energy utilization.

Therefore, stability analysis is required to ensure that the system can continue to operate normally under any disturbance, such as load variations, fault events, or sudden generation changes. Stability analysis is the study of a system to analyze its ability to remain in a stable condition, even after being subjected to disturbances. Furthermore, stability is necessary to maintain electricity quality standards and facilitate a smooth integration with the grid. Additionally, stability analysis aids in the best possible design and management of hybrid systems, allowing them to react to demand fluctuations or disturbances with minimal interruption and maximum energy efficiency. That’s why, a thorough understanding of stability analysis for HES is crucial to ensure the reliable and efficient design of these complex power systems, particularly given the challenges posed by their dynamic and multifaceted nature.

While HES definition, application, types, technologies, design considerations, and economics can be found in [14], it does not discuss the modelling, challenges, and stability assessment. The stability analysis in terms of transient and rotor angle stability is well explained in [6]. However, the authors do not mention other types of stability analysis. The design parameters of HESs, control and energy management within HESs are reviewed in [8], though the aspect of stability is not. Similarly, only the modelling of the HES system is explained in [15]. A thorough examination of stability concerns in HESs is provided in the review paper [16]. However, the recently added section on converter-based stability analysis, such as slow converter-driven stability and fast converter-driven stability, which is crucial given the growing importance of power electronic interfaces in contemporary hybrid energy systems, is not covered. Table 1 compares various aspects of HES stability covered in the existing literature with those addressed in the proposed work. It is evident that the existing literature provides individual insights, which together emphasize the need for the comprehensive approach presented in this paper.

In general, existing studies mainly focus on the mathematical modeling of renewable energy sources and usually neglect to address tge variety of stability analysis techniques that comes after modeling phase, including static, dynamic, small-signal, large-signal, and converter-based approaches. Therefore, a more thorough and integrated review that covers all stability assessment techniques is clearly needed. By offering a comprehensive overview of the stability analysis techniques relevant to HES, emphasizing their interdependencies, advantages, disadvantages, and applications, this work seeks to close that gap.

## 2. Hybrid Energy System and Its Characteristics

The word “hybrid” means the amalgamation of different elements or varieties. When several energy-conversion devices are combined and employed in tandem to meet an energy requirement, they are referred to as a hybrid energy system. A proper definition of HESs is beneficial, as they encompasses many options and are a crucial component of the multiplicity of energy conversion. Hence, HESs are defined by the University of Massachusetts, as “A combination of two or more energy conversion devices (e.g., electricity generators or storage devices), or two or more types of fuels for the same device, that, when integrated, overcomes limitations that may be inherent in either” [14].

### 2.1. Different Configuration of Hybrid Energy System

HES can be divided into three distinct configurations based on its features and manner of operation: central grid-connected hybrid system, isolated grid hybrid system, and special-purpose hybrid system. Each type is explained further.

**Figure 1:**
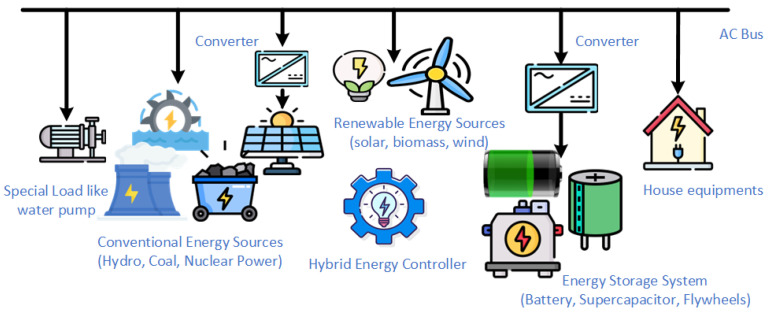
Structure of a basic HES system.

Central Grid-Connected Hybrid Systems: A central grid-connected HES streamlines design by allowing the central grid to control voltage and frequency. It indicates that different sources are synchronized with the primary grid. This minimizes the requirement for control components within the hybrid system. The utility grid typically delivers reactive energy and compensates for energy deficiencies or surplus generation by supplying additional energy or absorbing excess production [20]. A basic structure of the Central grid-connected HES system is shown in Figure 1.

Isolated Grid Hybrid Systems: Isolated grid HES configuration is independent of the central grid, meaning it has to maintain its frequency and voltage level. Critical functions such as supplying reactive power, controlling surplus energy from renewable sources to prevent system instability, and regulating frequency as well as voltage levels are also managed by these systems [21,22]. As the control strategy for the isolated system is quite complex, it becomes difficult to balance renewables and non-renewables without a central grid.

Special Purpose Isolated Hybrid Systems: Special-purpose HESs are utilized for specific purposes, such as heating, desalination, water pumping, military and disaster relief, aviation and marine applications [23]. They are not frequently integrated into a larger distribution network. They are an essential solution for focused and sustainable energy distribution when traditional systems are ineffective or unavailable. These systems typically avoid problems with managing excess power or regulating voltage and frequency. The conventional generators are added as a backup in cases where renewable energy sources are momentarily unavailable [14].

Despite the type of HES, they all have similar components; only their way of connection is different. HES components are explained further in the next subsection.

### 2.2. HES Components

HES is constructed by combining technologies such as rotating electrical machines, renewable energy generators, fossil fuel generators, energy storage devices, converters, loads, and controllers. HES exploits the advantages of conventional and renewable energy sources by combining them, creating a well-rounded strategy that improves grid stability, meets peak demand, and manages the intermittent nature of renewables. By facilitating a more adaptable and robust energy framework, this collaboration opens the door to a sustainable transition while preserving energy security. There are various components which are required to form HES, and those are mentioned below.

#### 2.2.1. Conventional Energy

Conventional energy comes from fossil fuels, such as coal, oil, and natural gas, and has served as the foundation for power generation worldwide [24]. Conventional energy sources are essential for maintaining stability and supply continuity in HES, especially when renewable energy sources like solar and wind are absent or insufficient. Conventional energy inherently maintains stability in a system as it has its inertia. These devices ensure a consistent and reliable energy supply.

#### 2.2.2. Renewable Energy

Renewable energy generators are energy sources that produce electricity from sustainable and natural sources [25]. Sources such as solar [26], wind [27], biomass [28], fuel cells, hydroelectric generators, etc., are examples of renewable energy. As they supply clean energy and also support traditional energy sources, these sources are crucial for HES formation. Wind energy is efficient in windy areas. Likewise, hydroelectric generators are found in areas with rivers or dams and biomass generators in rural areas. Although fuel cells are less popular, they are very efficient and do not affect the environment [29]. A review of hybrid renewable energy is also presented in [15].

#### 2.2.3. Energy Storage

In HES, energy storage systems are used to store excess energy and are also used as backup to provide energy at peak hours. It also performs vital tasks such as system regulation and load balancing. Depending on the system’s requirements, technologies such as batteries, flywheels, and thermal storage, each having distinct functions and advantages, are utilized [30]. These technologies stabilize variations in renewable generation and improve energy reliability. HESs can optimize the use of renewable energy sources, reduce the reliance on fossil fuels, and guarantee a consistent and reliable energy supply by combining these storage components [2]. Hybrid energy storage systems are discussed in [31].

#### 2.2.4. Converters

Power converters such as AC-DC, DC-DC, and DC-AC are necessary interfacing devices used for the integration of renewable energy sources and energy storage. Among these converters, DC-AC inverters are compatible with the main grid and transform DC electricity from renewable sources (such as solar panels) into AC power, as the main grid is AC in nature. DC-DC converters also modify the voltage levels between the system and storage devices. Batteries and other storage devices can be charged from AC power sources using AC-DC converters. These converters guarantee optimal energy flow and system stability while facilitating the smooth integration of many energy components (grid, storage, and renewables) [32].

#### 2.2.5. Energy Consuming Devices

Energy-consuming devices are appliances that use mechanical, thermal, or electrical energy to perform desired tasks. Generally, household appliances, industrial appliances, and power electronic devices use electrical energy to operate [33]. Further, there is a dump load that is essential for preserving system stability in hybrid energy systems (HES), particularly in isolated systems [14]. Dissipating excess energy when generation surpasses consumption aids in power balancing.

#### 2.2.6. Controllers

Although HESs have many advantages, they also have several technical issues, especially when it comes to system power quality. These difficulties include power fluctuations brought about by plug-and-play renewable energy systems (RESs) or the addition of more renewable energy sources [8]. Additionally, when switching between grid-connected and stand-alone modes, there may be variations in voltage and frequency. To alleviate power quality issues and ensure reliable, stable, and high-quality power output, HESs must be equipped with sophisticated control techniques [9].

The integration of these components into the grid is a very important aspect of building a hybrid energy system. The combination of these many technologies raises stability concerns. Stability analysis helps keep the system stable, so the overall system remains in conjunction and can work together to meet the electricity demand.

This paper is organized as follows. Section 2 presents the fundamentals and key features of HESs, highlighting the various distinct configurations for distinct purposes and different components involved in their design. Section 3 provides a comprehensive understanding of various methods of stability analysis applicable to HESs, while Section 4 discusses the associated challenges regarding maintaining system stability. Finally, conclusions are presented in Section 5.

## 3. Stability Analysis

Stability in power systems is defined as “the ability of an electric power system, for a given initial operating condition, to regain a state of operating equilibrium after being subjected to a physical disturbance, with most system variables bounded so that practically the entire system remains intact” [34]. Stability assessments are carried out to determine a system’s ability to sustain steady-state functioning after minor or major disruptions. Also, it is necessary for numerous contingency situations, including faults, load changes, brownouts, and blackouts. Numerous instances have occurred in the past [35] indicating that the stability analysis of energy systems is important, as it has been observed that many incidents could have been prevented with adequate system understanding and situational awareness [36]. Offline and online stability analyses of the system are necessary to address stability issues. Offline research is done during the system’s design phase before development. After that, an online stability study is conducted to monitor the system and make it stable. Data gathering, using tools like sensors and phasor measuring units, is a crucial stage in online analysis. Collected data such as voltage, current, frequency, and phasor angle aids in determining the system’s next state and preserving equilibrium [37].

Due to the integration of complex and renewable energy sources, HESs face significant challenges in performing accurate and real-time stability analysis. However, the availability of high-resolution sensor data, combined with the rapid advancements in artificial intelligence (AI) techniques, enables faster, data-driven stability assessments, enhancing both system reliability and operational efficiency. This draws attention to the shortcomings of conventional techniques for handling the dynamics of nonlinear, high-dimensional, and unpredictable systems. To assess their contributions to defect detection, real-time monitoring, predictive maintenance, and control optimization, the authors categorize and examine AI techniques such as machine learning, deep learning, fuzzy logic, expert systems, genetic algorithms, and reinforcement learning. In [38], a thorough comparison of AI techniques, real-world case studies, and a research roadmap that highlights the necessity of hybrid and scalable AI systems in contemporary power systems are explored. The authors of [39] assess the benefits and drawbacks of several AI models, including CNNs, ANNs, SVMs, and LSTMs, in forecasting grid stability in diverse contexts. Most significantly, their study presents a new idea called Converter-Dominated Power System State of Stability, which integrates converter control characteristics (such as damping, droop coefficients, and virtual inertia) into stability evaluation.

**Figure 2:**
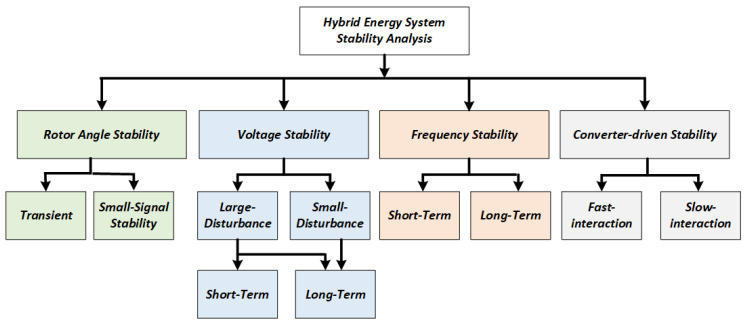
Types of stability.

In power system stability analysis, the time span plays a critical role in defining the type of stability problem and the corresponding analysis required. Typically, stability analysis is performed over time spans ranging from seconds to a few minutes following a system disturbance. Moreover, it is essential to model the system appropriately based on the study time span [40], as the modelling approach varies depending on the specific type of stability analysis being conducted. Different phenomena occur at different time scales, such as short-term stability over seconds or long-term stability over hours. The results of stability analysis help in customizing control strategies, ranging from quick fixes for disruptions to long-term stability planning. Reliable and effective power system operations and management are ensured by the time-span consideration. After a time span, the intensity of disturbance also affects the stability type. In case of a small disturbance, a small-signal analysis is performed. If the disturbance is large, transient stability analysis is conducted. Figure 2 describes different types of dynamics of stability analysis based on different time spans.

**Figure 3:**
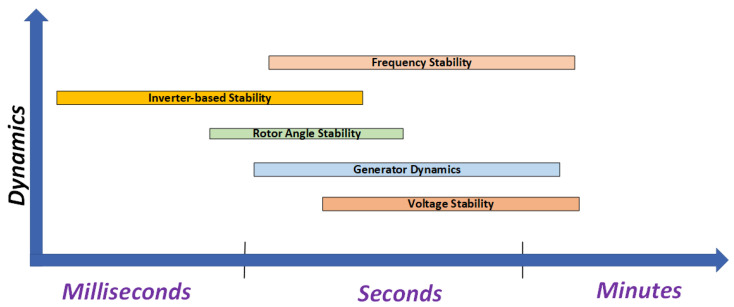
System dynamics involved in stability Analysis based on Time span.

There are different types of stability analysis for power systems based on time span and intensity, which can be applied to HES [34]. In 2004, the IEEE/CIGRE Joint Task Force on Stability Terms and Definitions [34] categorised stability analysis into three groups, namely rotor angle stability, voltage stability, and frequency stability analysis. However, in 2016, due to the emergence of power electronics technologies and their growing utilisation for the integration of renewable energy, energy storage and power electronic loads, the committee added a new category called converter-driven stability analysis [41]. Because of the converters, the power system’s dynamic reaction is increasingly relying on the quick response of the power electronic devices, which modifies the system’s dynamic response. That’s why a new category of stability analysis is required. Figure 3 lists all categories of stability studies required for HES. Even though all forms of stability are linked and lead to one another, it is crucial to examine each independently to have a proper understanding.

Each type of stability study is discussed in detail in the following section.

### 3.1. Rotor Angle Stability

The ability of synchronous machines (such as motors and generators) in HES to stay in sync with each other after following a disturbance, like a fault or abrupt shift in load, is known as rotor angle stability. It guarantees that the machines continue to run at a steady frequency and phase relationship, which is an essential component of power system stability [42]. When there is a disturbance or change in the power balance within the system, the synchronous machine’s rotor angle δ may diverge from its equilibrium position. The machine may get unsynchronized, and the system may experience system-wide failures if the rotor angle of the machine diverges too much. A synchronous machine’s rotor angle (δ) has a dynamic behaviour concerning the system, which can be observed from the swing equation, i.e., the basis equation regulating rotor angle stability with time. A non-linear analysis needs to be conducted since the system does not function linearly during periods of significant disturbance. The rotor dynamics are governed by the swing equation as shown in Equation (1). It shows the relationship of the acceleration of the rotor angle δ to the difference between mechanical power input $P_{m}$ and electrical power output $P_{e}$. Electrical power output is typically expressed in terms of system voltages and reactance as shown in Equation (3), influencing the rotor’s stability.

The swing equation is a second-order differential equation that relates the electrical power, mechanical power, and inertia of the machine. The swing equation is given by:

(1) $$\;\frac{2H}{\omega_{s}}\frac{d^{2}\delta }{dt^{2}}=P_{m}-P_{e}\;$$

where:

- *H*: Per-unit inertia constant of the machine (in seconds).
- $\omega_{s}$: Synchronous angular velocity of the system (in radians per second), typically 2πf, where *f* is the system frequency (e.g., 50 Hz or 60 Hz).
- δ: Rotor angle of the synchronous machine (in radians), representing the angular displacement between the rotor and the synchronously rotating reference frame.
- $P_{m}$: Mechanical input power to the generator (in per unit or MW).
- $P_{e}$: Electrical output power of the generator (in per unit or MW).
- $\frac{d^{2}\delta }{dt^{2}}$: Angular acceleration of the rotor (in radians per second squared).

The swing equation Equation (1) is often expressed in a simplified form of per-unit:

(2) $$\;M\frac{d^{2}\delta }{dt^{2}}=P_{m}-P_{e}\;$$

where:

$$M=\frac{2H}{\omega_{s}}$$

is the inertia constant (in per unit seconds).

The electrical power output $P_{e}$ is given by:

(3) $$\;P_{e}=\frac{V_{1}V_{2}}{X}\sin\left(\delta \right)\;$$

where:

- $V_{1}$ and $V_{2}$: Voltages at the sending and receiving ends of the transmission line (in per unit or kV).
- *X*: Reactance of the transmission line (in per unit or ohms).
- δ: Rotor angle (in radians).

Substituting $P_{e}$ into the swing equation to get the final equation for rotor angle dynamics:

(4) $$\;M\frac{d^{2}\delta }{dt^{2}}=P_{m}-\frac{V_{1}V_{2}}{X}\sin\left(\delta \right)\;$$

This nonlinear differential equation describes the dynamics of the rotor angle and is the fundamental equation for analyzing rotor angle stability. Based on the magnitude of the disturbance, the rotor angle stability analysis is divided into two primary types: Transient stability analysis and small signal stability analysis.

#### 3.1.1. Transient Stability Analysis

Transient stability analysis involves a large-disturbance analysis of the system. It is essential to evaluate a system’s ability to maintain synchronism following significantly large disturbances, such as short circuits, sudden load changes, or generator outages. This analysis focuses on the system’s dynamic response over a few seconds after the disturbance, particularly on the rotor angle stability of synchronous generators.

**Figure 4:**
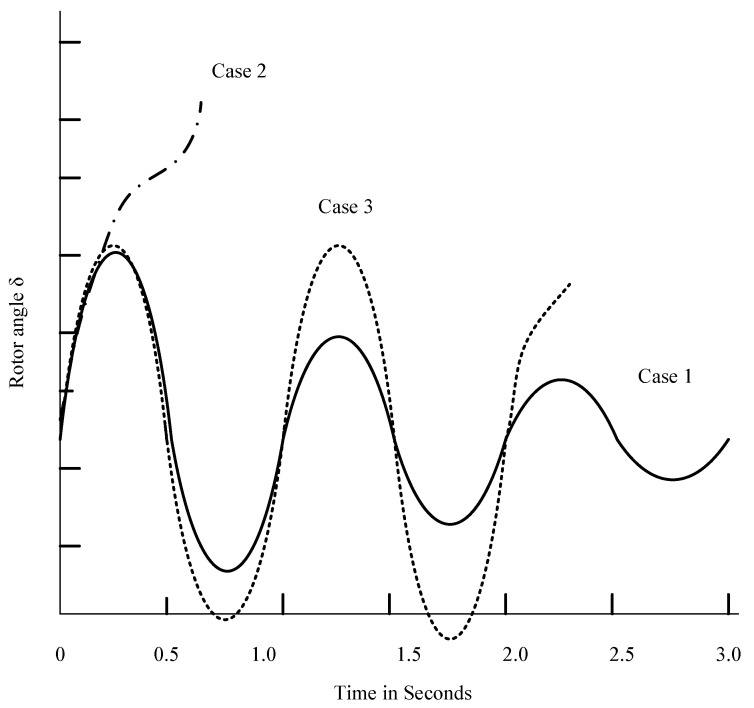
Rotor Angle deviation in different cases.

In Figure 4, the behaviour of synchronous machine rotor angle is shown for stable and unstable conditions. In case 1, the rotor angle reaches its greatest value, then falls and oscillates with a diminishing magnitude until stabilising. In case 2, synchronisation is lost and the rotor angle sharply increases. First-swing instability is the term for this kind of instability. In case 3, the rotor angle remains steady during the initial swing before becoming unstable due to increasing oscillations. This leads to the small-single instability.

**Figure 5:**
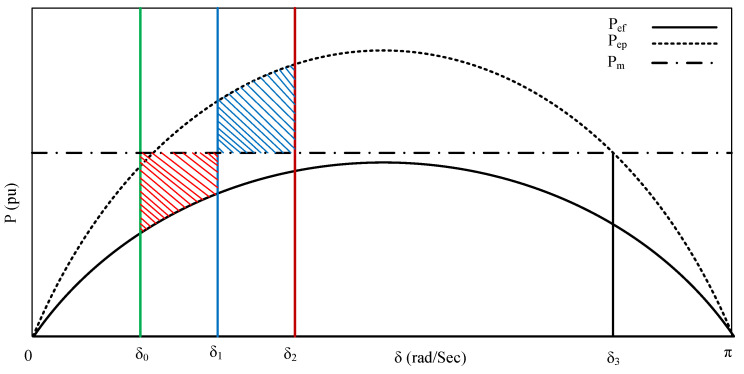
Equal area criteria; Red & blue color area represents accelerated power & decelerated power respectively.

The above-explained time-dependent differential equation, Equation (1) is used to do the transient analysis. This equation is solved numerically using techniques such as step-by-step integration or the Runge-Kutta method. In light of the uncertainties associated with renewable power generation, the work [43], has proposed a methodology for examining the initial swing rotor angle stability of power systems. Another popular method for transient stability analysis is the Equal Area Criterion (EAC), which is a graphical approach for assessing stability in single-machine infinite bus systems [44]. The analysis involves studying pre-disturbance conditions, the system response during disturbances, and recovery after clearing faults. EAC diagram is shown in Figure 5. $P_{ef}$ is electrical power during the fault, $P_{ep}$ is electrical power post fault. $\delta_{0}$ is the initial rotor angle i.e., at the time of the fault, $\delta_{1}$ is the angle at fault clearing, $\delta_{2}$ is the maximum swing for the rotor, and $\delta_{3}$ is the maximum permissible rotor angle after the fault. The Area covered by red is the accelerated power, and the area covered with blue is the decelerated power. When accelerated power is equal to the decelerated area, the system’s stability is ensured. A critical parameter in this analysis is the Critical Clearing Time (CCT), representing the maximum allowable time to clear a fault without losing synchronism.

Other ways of conducting transient stability are the Lyapunov-based approach, hybrid approach, probabilistic approach and AI-based approach. The Lyapunov approach is based on Lyapunov’s second (or direct) method, a mathematical stability theory that assesses system stability without knowing the precise solution of the system’s differential equations by using a particularly designed scalar function known as a Lyapunov function. It can estimate stability margins and stability regions. Though they assess system stability without temporal integration, they face challenges in simulation. A hybrid approach combines several methods to improve accuracy, minimize computing load, and offer more thorough insights into rotor angle stability. Furthermore, data-driven approaches are becoming more and more popular for real-time transient stability prediction. While they provide quick findings, they have drawbacks such as poor interpretability and susceptibility to topological changes. A more realistic evaluation is offered by probabilistic techniques for rotor angle stability analysis, which considers uncertainties in system characteristics such as failure scenarios, load variations, and renewable generation. Especially in contemporary grids with significant stochastic resource penetration, this improves system planning and permits risk-informed decision-making. Every technique has advantages and disadvantages for real-world applications in contemporary power systems. Table 2 provides a comparison of different analysis methods for transient stability.

Transient stability analysis is vital in designing protection systems, evaluating the response of the system under faults, and optimizing controllers to ensure that hybrid energy systems operate reliably and maintain stability under dynamic conditions.

#### 3.1.2. Small Signal Stability Analysis

The ability of a power system to remain synchronised regardless of minor disruptions is known as small signal stability. These disturbances are usually small variations in load, generation, or system setup. In this analysis, the linearized behaviour of the system around its operating point is the main focus of the analysis. Linearization is carried out with the help of the Taylor series expansion, where the higher-order nonlinear terms are neglected and sin(δ)∼δ, as mentioned in Equation (7).

The linearized swing equation forvsmall signal stability study of synchronous machine is given by:

(5) $$\;\frac{2H}{\omega_{s}}\frac{d^{2}\delta }{dt^{2}}+D\frac{d\delta }{dt}+\Delta P_{e}=\Delta P_{m}\;$$

The electrical power and angle relationship is given by:

(6) $$\;P_{e}=\frac{V_{1}V_{2}}{X}\sin\left(\delta \right)\;$$

For small perturbations, sin(δ)≈δ, so:

(7) $$\;\Delta P_{e}=K_{s}\delta \;$$

where $K_{s}$ is the synchronizing power coefficient.

(8) $$\;K_{s}=\frac{V_{1}V_{2}}{X}\;$$

Substituting $\Delta P_{e}$ into the swing equation:

(9) $$\;\frac{2H}{\omega_{s}}\frac{d^{2}\delta }{dt^{2}}+D\frac{d\delta }{dt}+K_{s}\delta =\Delta P_{m}\;$$

Many studies related to grid-connected synchronous machine’s small signal stability under different combinations of HESs are discussed in detail in [49,50,51]. For stability studies, the swing equation can be expressed in state-space form:

(10) $$\;\dot{x}=Ax+Bu\;$$

where:

- *x*: State vector (e.g., rotor angle δ and speed ω).
- *A*: State matrix, determines system eigenvalues.
- *B*: Input matrix.
- *u*: Input vector.

Small signal stability analysis is done with the help of eigenvalues of the state matrix, *A* [52]. Eigenvalues are crucial for small-signal stability analysis and power system control design because they offer a quantitative assessment of stability and oscillatory behaviour. By suggesting a way to combine different dynamic network models and dynamic device models to create a state space model of a Hybrid system, the work in [53] offered a novel contribution.

### 3.2. Voltage Stability

**Figure 6:**
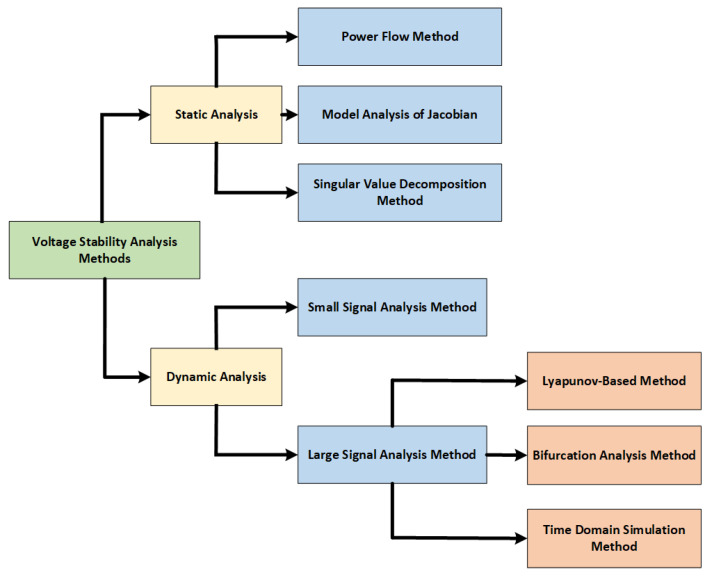
Voltage Stability Analysis Method [55].

Voltage stability study is crucial for assessing a power system’s ability to maintain acceptable voltage levels under normal conditions and after disturbances. The ability of a power system to maintain constant voltages at all buses in the system following a disturbance from a particular starting operating condition is known as voltage stability [34]. It focuses on the system’s ability to supply reactive power and manage load demands without experiencing uncontrollable voltage drops or rises. Voltage instability typically arises from a mismatch between the reactive power supply and demand. The gradual decline in some buses’ voltages leads to voltage instability. Rotor angle instability may also lead to a progressive decline in bus voltages. The same set of nonlinear dynamic equations (Equation (1) to Equation (9)), which are derived from the Swing equations for rotor dynamics and the load flow equations for voltage and power flow, govern both voltage stability and rotor angle stability [54]. Other factors leading to voltage instability are heavy load on transmission lines, insufficient reactive power, low source voltage and voltage sources situated away from the load dispatch centre [40]. In addition to rotor angle stability, the HES components, i.e., loads, generators and their exciter, automatic generation control and static var systems, can also impact the voltage stability. Although progressive bus voltage drops are the most prevalent type of voltage instability, overvoltage instability is still possible. Voltage stability is divided into small and large disturbances, same as rotor angle stability. However, it is analyzed using static and dynamic methods. Different techniques for both methods are shown in Figure 6 extracted from [55].

#### 3.2.1. Static Analysis

The capacity of the system to sustain constant voltage levels at every bus in steady-state operating conditions, free from time-dependent disruptions, is known as static voltage stability [56]. Static analysis examines the steady-state relationship between load and voltage, often employing tools such as power-flow studies or P-V (Power–Voltage) and V-Q (Voltage–Reactive Power) curves, Jacobian model analysis, or the singular value decomposition method. The curves help identify critical operating points, such as maximum load capacity or voltage limits [57]. The P-V and V-Q curves analyze the relationship between active power (P) and voltage (V) and reactive power (Q) and voltage (V), respectively; these techniques are often used in investigating static stability. These techniques aid in locating instances of voltage instability, which usually occur as load or reactive power demand gradually increases. This study works best in utility-connected systems since it functions under steady-state settings [58].

#### 3.2.2. Dynamic Analysis

The dynamic analysis considers the time-dependent behaviour of voltage following disturbances, such as generator outages or sudden load changes, and requires solving differential equations representing system dynamics. Key measures include ensuring sufficient reactive power sources, deploying voltage regulators, and designing controllers, such as Flexible AC Transmission Systems (FACTS) [18]. Different methods are used to evaluate dynamic voltage stability. In order to analyze eigenvalues and gain insight into oscillatory behavior and control design, small-signal analysis linearizes the system around an operational point [59]. Fault recovery and inverter response studies frequently use time-domain simulation [55], which solves differential–algebraic equations and fully represents the system’s nonlinear and transient response. Techniques such as Lyapunov functions and energy function-based methods evaluate stability margins for large disturbance without the need for explicit time-domain solutions. Furthermore, crucial parameter thresholds are identified using bifurcation theory [60], where Hopf bifurcations indicate the beginning of oscillatory instability and saddle-node bifurcations indicate proximity to voltage collapse. These methods are especially crucial for contemporary power systems with substantial inverter-based resource penetration, when control dynamics and traditional inertia are minimal.

Voltage stability studies are vital for preventing voltage collapse, optimizing reactive power management, and ensuring the secure and reliable operation of HESs, particularly those incorporating renewable energy sources that are prone to variability. These analyses guide system design, maintaining stable voltage profiles even under challenging operating conditions.

### 3.3. Frequency Stability

The ability of a power system to maintain a constant frequency after a serious disruption that causes a large imbalance between load and generation is known as frequency stability. In other words, frequency stability is the capacity to minimize unintended load loss while maintaining or restoring equilibrium between system generation and load. The system may become unstable as a result of prolonged frequency swings that trip-generating units and/or loads. In HES, the converter is an integral component of integrating renewable energy, such as wind turbines and solar photovoltaics, with traditional sources. The converter, being a power electronic interface, has no rotational mass. Because of this, it lacks inertial features, making it challenging for frequency management. However, synchronous generators powered by conventional sources inherently encourage frequency stability.

Frequency stability analysis can be divided into short-term and long-term analysis depending on the time scales and dynamics involved in sustaining system frequency. These two aspects concentrate on distinct phenomena and elements of the system [34]. The ability of the system to sustain frequency within a few seconds right after a disruption is known as short-term frequency stability. On the other hand, the ability of the system to sustain frequency within a longer time following a disruption is known as long-term frequency stability.

Knowing that the frequency produced in an electric network is directly proportional to the generator’s rotation speed, the frequency control issue might be simply converted into a turbine-generator unit speed control issue. To ensure power system frequency stability, several frequency control loops are needed when HESs are designed, depending on the frequency deviation range. Frequency stability issues are typically linked to inadequate generating reserve, inadequate control and protection equipment coordination, or inadequate equipment reactions.

**Figure 7:**
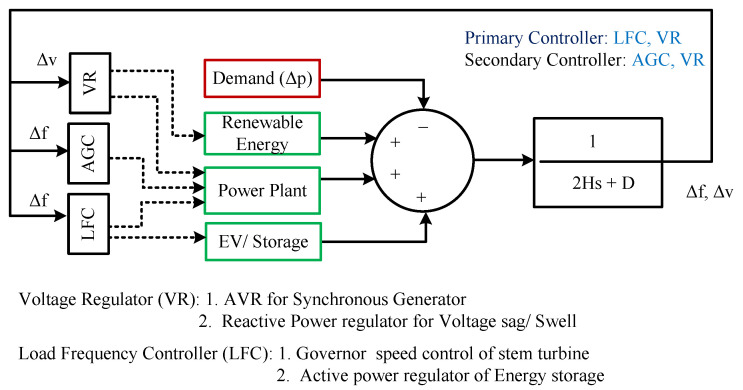
Primary and Secondary Frequency Control Loops.

There are three control loops to maintain frequency stability: primary control (droop control explained further in this section), secondary control, as shown in Figure 7, and tertiary control including coal, and hydropower as reserves. Under normal conditions, small frequency deviation can be controlled by the primary controllers which are local and automatic controllers such as governor response, and droop control take the primary actions within seconds. For off-normal conditions, secondary control, which is a centralized and automatic controller such as load frequency control, the energy reserve restores the frequency deviation. Load frequency control is an important control which maintains frequency by power exchange between the neighbourhood. However, for critical scenarios like faults, actions are controlled by tertiary controllers such as under-voltage load shedding, which works by mutual shedding of the load units [61]. Both short-term and long-term evaluations are essential for guaranteeing the reliability of a power system. Long-term analysis focuses on gradually restoring frequency and system balance, whereas short-term analysis deals with instantaneous dynamic reactions.

Primary controllers work for short-term disturbance and try to settle down the system to a stable position. On the other hand, secondary and tertiary controllers work When a long-term disturbance occurs.

Frequency and power relationship shows the interdependency between the variables. Droop control establishes a proportional relationship between the change in system frequency (Δf) and the change in active power output (ΔP) of a generator.

Mathematically:

(11) ΔP=−R·Δf

where:

- ΔP: Change in active power output.
- *R*: Droop coefficient or droop constant, given by:
  (12) $$\;R=\frac{\Delta f}{\Delta P}\;$$
- Δf: Deviation of system frequency from the nominal frequency.

The frequency-power characteristic’s slope is determined by the droop coefficient (*R*). A steeper slope, indicated by a lower *R*, indicates that the generator will react more forcefully to variations in frequency. Frequency stability analysis can be done with the help of various techniques such as control-based analysis, dynamic simulations [62], transient stability studies [63], and small-signal stability. In [63], large frequency disturbance cases are analysed, behaviours and models of various power system elements are discussed, and measures to improve frequency stability are mentioned. The hybrid system of solar, battery, and biomass that is experiencing frequency loss due to a lack of inertia has been examined in [62]. They have provided a solution and verified it in a real-time simulator. The study in [64], investigates wind turbines which can help control the system’s frequency in islanded mode and even prove that it can behave as a primary frequency controller. With machine learning to develop an adaptive controller, frequency control activity can be improved, although it can be more complex [65].

Techniques used in HES include energy storage systems for quick responses to stability problems, grid-forming inverters that mimic inertial behaviour, and demand response to balance supply and demand. Even in weak or isolated grids, stability is further improved by sophisticated hybrid control schemes and predictive algorithms, which guarantee dependable and effective operation. With the help of contemporary control technologies and the advantages of both conventional and renewable energy, HES can provide reliable frequency management in dynamic environments.

### 3.4. Converter-Driven Stability

This stability adds a novel dimension to the stability analysis. It requires new control techniques and studies. It becomes crucial to include converter-driven stability analysis as electronic-based power sources and storage, and loads are getting integrated more into the power system. A thorough analysis of the control dynamics and stability issues arising from converter-interfaced generation highlights the importance of converter-driven stability. The stability dynamics of power networks have been profoundly changed by the growing integration of renewable energy sources (RESs) such as wind and solar PV, which are interfaced through power electronic converters. It detaches generators from grid dynamics, which results in issues such as decreased reactive power reserves, limited fault current contribution, and decreased synchronous inertia. Rotor angle stability, frequency stability, and voltage stability are all adversely affected by these modifications. Furthermore, because of their high-bandwidth control loops, converters can cause oscillatory instabilities and induce sub-synchronous interactions. While reduced inertia and dynamic responses during faults impair transient stability, improperly tuned PLLs or inadequate dampening in converter control might jeopardize small-signal stability [66]. Additionally, unstable converter control might result from poor grid circumstances under both grid-following and grid- forming modes. The primary problems in dynamic modelling and stability analysis for converter-based systems are discussed in this study [67], which also provides an overview of the state of the art in this area. All stability issues and phenomena resulting from couplings between the dynamic control loops of converter-interfaced generation and the electromechanical or electromagnetic dynamics of power systems or converter-interfaced generation components are included in the term converter-driven stability [68]. This leads to the bifurcation of this stability based on the time scale of system response into two different categories: slow converter-driven stability and fast converter-driven stability.

Slow Converter-driven Stability: The phenomena that take place within the fundamental grid frequency (f = 50 Hz) are included in slow converter-driven stability. These are referred to in the literature as sub-synchronous oscillations (less than 50 Hz), sideband oscillations (around the fundamental frequency) [69], or low-frequency oscillations (less than 10 Hz in [70]). The reasons for this kind of instability depend on the inertia of the grid, Phase lock loop (PLL), and voltage and power control. To effectively analyse the power–voltage interaction between the converters and the grid, study [71] suggests a harmonic power model for converter-based networks.

Fast Converter-driven Stability: This stability occurs within frequency ranges from below 100 Hz to kHz. As a result, numerous instability events with very diverse sources are covered. Interactions brought on by inner current control loops or control delays are the main drivers of fast converter-driven instability effects. Harmonic instability issues may arise as a result of the converter switching activities, which affect the resonance frequency and have the ability to move it to critical frequency ranges [72]. One of the primary causes of harmonic instability issues is converter control delays. The control delay can interact with the LC resonance frequency [73]. High-frequency oscillations and issues with harmonic instability might result from interactions between the converter’s inner control loops and passive grid components.

The methods of analysis for this stability are divided into small-signal analysis and large-signal analysis, and cane be further analysed in the time and frequency domains. For small-signal analysis in the time domain, the system is linearised around an operating point, and a dynamic response to small perturbations in the simulation environment is observed. Small-signal stability problems are best suited for time-domain analysis based on state-space models. In the frequency domain, the small-signal model is transformed to analyse control-loop performance using tools such as Bode plots, Nyquist criteria, and impedance-based methods. This makes it possible to evaluate eigenvalues, damping ratios and interactions of converter control, such as Phase-Locked loops (PLLs) and controller loops. These converters perform switching operations which introduce harmonic components across a wide frequency range.

**Figure 8:**
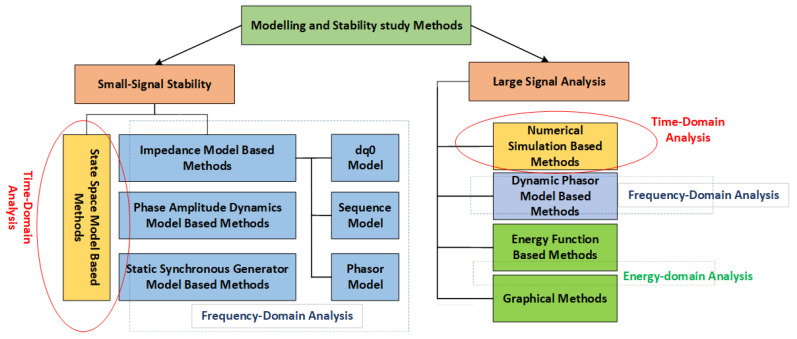
Methods to study converter-driven stability.

The goal of converter-based large-signal stability analysis is to assess how converters react to extreme disruptions like faults, load rejections, or abrupt generation disconnection. Nonlinear simulations, which solve the system’s differential–algebraic equations to observe voltage, current, and frequency trajectories following a disturbance, are used to evaluate large-signal behaviour in the time domain. Harmonic balance, description functions, and nonlinear frequency response functions are occasionally used to estimate the response of converters under periodic or quasi-periodic disturbances in the frequency domain, even though standard small-signal linearization loses some of its validity. But compared to time-domain simulations, frequency-domain large-signal analysis is typically more constrained and less useful, particularly for systems that exhibit nonlinear and fast-switching behaviour. In general, time-domain approaches continue to be the most dependable for examining converter-dominated large-signal dynamics; but, hybrid approaches that incorporate time-frequency insights are becoming more popular in order to handle the complexity of contemporary power systems [74,75]. Methods of converter-based analysis are shown in Figure 8 [67]. Methods to help choose the best strategies for resolving converter-driven stability issues are presented in [19]. To have an overall idea, a comparison of different stability analysis techniques with pros and cons is shown in Table 3.

## 4. Challenges in Stability Analysis

Various energy sources, including solar, wind, diesel generators, and energy storage devices, are combined in HESs to produce dependable and sustainable power. Because of the intermittent nature of renewable energy sources and the dynamics brought about by power electronic converters, these systems are facing more stability issues as the integration of renewable energy is increasing. Reducing inertia, controlling frequency and voltage, and implementing complex control algorithms are all necessary to guarantee system stability. For HES to operate dependably and robustly, the following issues must be addressed.

1. Modelling and control design: Any system must have an accurate system model to do stability analysis, which is a challenging task. Due to the lack of very precise models for a few components, it is a tedious operation. Under some circumstances, the system modelling choice might not be appropriate in practical situations [76]. The system’s control portion is increasingly complicated as renewable energy sources proliferate [77]. It is challenging to consider every factor at once when doing stability analysis.
2. Intermittency of Renewable Energy: Conventional generating units frequently find difficulties in adjusting during high-stress abrupt and frequent start-ups, along with quick net load fluctuations. It is because of RES’s intermittent and variable nature [78]. It is mostly dependent on weather parameters for generating power, and that’s why its prediction becomes difficult. This variability has varying effects on system operation and planning over different timescales. During long-term resource planning, changes in net load have little effect. In day-ahead operational planning, daily cycles become crucial. To preserve system stability and guarantee dependable grid operation, control systems must react quickly to sudden variations in RES production, which are sometimes measured in milliseconds.
3. Lack of Inertia: There is a major role of inertia in the system to maintain flexibility and frequency stability. Due to the integration of more renewables, which are non-synchronous devices, the grid will face an overall decrease in conventional inertia. This will cause an increase in frequency fluctuations, and subsequently, major fluctuations cause major instability in the system. The study [79] examines the crucial role inertia plays in preserving grid flexibility and stability, emphasising the difficulties brought by low inertia as a result of the integration of renewable energy. There is also a discussion of suggested remedies to deal with these problems.
4. Grid Integration and Load Balancing: One essential component of power system networks is the incorporation of renewable energy sources in islanding mode. Connecting these sources to power networks produces several difficulties because of their unpredictable and variable nature. The operation of grid-connected renewable energy sources during small voltage drops and inter-area oscillation is challenging and studied in [13]. It includes stability problems resulting from power transfers across various grid zones. Addressing these issues is essential in preserving overall grid stability and resiliency.
5. Security Concern: Use of machine learning for energy forecasting of renewables such as solar and wind, and internet-of-things (IoT) devices for monitoring and data collection forms an interconnected web environment which generates a lot of data making it vulnerable to security breach [80]. Other important areas of risk include the communication networks, control systems, and research data. The overwhelming amount of data produced by these networked devices emphasises how urgently strong cybersecurity safeguards are needed. The stability, effectiveness, and resilience of contemporary power grid operations depend on safeguarding control systems, ensuring the security and integrity of data flows, and reducing cyber threats.
6. Power Quality: Renewable energy, along with conventional sources, has become a key contributor in balancing the load supply demand in modern society. There are some standards related to the power quality, which is being received by the consumers. Because of the intermittent nature of these sources, there are a lot of fluctuations in the voltage and frequency, leading to a decrease in power quality. The work in [81], is divided into two major sections, one reviews the literature in great detail on new issues related to power quality, and another recommends some solutions to deal with these power quality issues.

## 5. Conclusions

To assist the integration of renewable energy sources, encourage the adoption of HESs, and accomplish sustainability objectives, stability analysis advancements are essential. The integration of various energy sources, dynamic interactions, and time-dependent behaviours presents serious stability issues for HESs. Rotor angle analysis, voltage stability analysis, frequency stability analysis, and converter-driven stability procedures are important methods to guarantee stability in hybrid energy systems. When combined, these techniques provide a thorough foundation for preserving reliable and regular system performance in the case of fluctuations and disruptions. Although they require high-quality data, emerging artificial intelligence-based methods offer flexible solutions. However, sensors that gather data in real-time are capable of capturing high-quality data for conducting these kinds of studies. An effective evaluation of the rotor angle, voltage, frequency, and converter-driven stability is made possible, which permits quick and thorough stability assessments by facilitating an accurate model. These sensors provide an accurate detection of disruptions and dynamic changes in the system by providing real-time data on voltage, current, frequency, and phase angles, making it possible to conduct HES stability analysis. This study thoroughly examines several methods for carrying out stability analysis; these are discussed in this paper in detail within the framework of HESs. Our study also draws attention to their importance and the difficulties faced during stability analysis for HESs. It highlights the necessity of creative methods to improve system stability and the significance of continuous research to maximise HESs’ functionality and dependability. Together, these techniques aid in resolving stability issues, allowing HESs to support more resilient and sustainable energy systems.

Looking ahead, the future of HES stability depends on the creation of intelligent, self-adaptive systems that can respond dynamically to real-time conditions. Prediction accuracy and interpretability will be enhanced by combining physics-informed models with machine learning and deep learning methodologies. Furthermore, mitigating vulnerabilities brought on by cyber-threats or communication breakdowns will require integrating cyber–physical resilience into stability frameworks. A virtual platform for simulating and optimising stability under various operating circumstances may be made available by the development of digital twins for HESs. In addition to improving HESs’ dependability, these upcoming developments will encourage widespread implementation in intelligent and sustainable energy systems.

## Figures and Tables

**Table 1:** Comparison of key aspects covered in the HES literature.

Aspect Covered	[6]	[14]	[16]	[17]	[18]	[19]	Proposed Paper
HES Definition	×	✓	×	✓	✓	×	✓
Stability Classification	×	×	✓	✓	×	×	✓
Roto Angle & Transient	✓	×	✓	✓	✓	×	✓
Voltage Stability	×	×	✓	✓	✓	✓	✓
Frequency Stability	×	×	✓	✓	✓	✓	✓
Converter-Based Stability	×	×	×	✓	✓	×	✓
Renewable Component	✓	✓	✓	✓	✓	✓	✓
Non-Renewable component	✓	✓	×	✓	✓	×	✓
Stability Issues	✓	×	✓	✓	✓	✓	✓
Stability Analysis Methodologies	✓	×	×	×	✓	✓	✓

**Table 2:** Comparison of different analysis methods for transient stability.

Method	Type of Analysis	Accuracy	Speed	References
Time-Domain Analysis	Numerical	High	Slow	[45]
Equal Area Criteria	Graphical	Basic	Fast	[44]
Direct Method	Analytical	Medium	Fast	[46]
Hybrid Method	Analytical	High	Fast	[45]
AI-based Methods	Data-Driven	Varies	Very Fast	[47]
Probabilistic Method	Statistical	High	Medium	[48]

**Table 3:** Comparison of stability analysis methods for HES.

Analysis Type	Focus	Application in HES	Advantages	Limitations
Static Stability	Small perturbations under steady-state	Voltage margin, loadability	Simple, fast	Ignores dynamics
Dynamic Stability	System response over time	Time-domain response in microgrids	Captures transient effects	Computationally intensive
Small-Signal Stability	Linear response near operating point	Inverter control, weak grid conditions	Useful for control design	Assumes linearity
Large-Signal Stability	Nonlinear behavior under major disturbances	Fault ride-through, fault stability	Realistic for faults	Complex simulation
Frequency Stability	Maintaining system frequency	Frequency support in islanded systems	Important for low-inertia HES	Sensitive to controller modeling
Voltage Stability	Maintaining voltage levels	Voltage support from hybrid sources	Identifies weak nodes	May miss global impacts
Converter-Based Stability	Power electronic control dynamics	Analysis of converter interactions	Captures fast dynamics	Requires detailed modeling
Probabilistic Stability	Stability under uncertain scenarios	Uncertainty in load, renewables	Captures variability	High computational cost
AI/Data-Driven Stability	Stability prediction using data	Real-time monitoring and control	Fast, scalable	Needs large datasets

## Data Availability

No new data were created or analyzed in this study. Data sharing is not applicable to this article.
